# Cost-effectiveness of intrapartum azithromycin to prevent maternal infection, sepsis, or death in low-income and middle-income countries: a modelling analysis of data from a randomised, multicentre, placebo-controlled trial

**DOI:** 10.1016/S2214-109X(24)00517-5

**Published:** 2025-03-26

**Authors:** Jackie K Patterson, Simon Neuwahl, Sydney Kirsch, Janet L Moore, Alan T N Tita, Waldemar A Carlo, Adrien Lokangaka, Antoinette Tshefu, Musaku Mwenechanya, Elwyn Chomba, Avinash Kavi, Mrityunjay C Metgud, Shivaprasad S Goudar, Richard J Derman, Poonam Shivkumar, Manju Waikar, Archana Patel, Patricia L Hibberd, Paul Nyongesa, Fabian Esamai, Osa A Ekhaguere, Sherri Bucher, Saleem Jessani, Shiyam S Tikmani, Sarah Saleem, Blair J Wylie, Robert L Goldenberg, Sk Masum Billah, Ruth Lennox, Rashidul Haque, William A Petri, Manolo Mazariegos, Nancy F Krebs, Jennifer J Hemingway-Foday, Denise Babineau, Marion Koso-Thomas, Elizabeth M McClure, Melissa Bauserman

**Affiliations:** a University of North Carolina at Chapel Hill, Chapel Hill, NC, USA; b RTI International, Research Triangle Park, NC, USA; c University of Alabama at Birmingham, Birmingham, AL, USA; d Kinshasa School of Public Health, Kinshasa, Democratic Republic of the Congo; e University Teaching Hospital, Lusaka, Zambia; f Women's and Children's Health Research Unit, KLE Academy of Higher Education and Research, Jawaharlal Nehru Medical College, Belagavi, India; g Thomas Jefferson University, Philadelphia, PA, USA; h Mahatma Gandhi Institute of Medical Sciences, Sevagram, India; i Government Medical College, Nagpur, India; j Lata Medical Research Foundation, Nagpur, India; k Datta Meghe Institute of Medical Sciences, Wardha, India; l Boston University School of Public Health, Boston, MA, USA; m Department of Child Health and Paediatrics, Moi University School of Medicine, Eldoret, Kenya; n Indiana School of Medicine, Indiana University, Indianapolis, IN, USA; o Aga Khan University, Karachi, Pakistan; p Columbia University School of Medicine, New York, NY, USA; q The International Center for Diarrheal Disease Research, Dhaka, Bangladesh; r LAMB Hospital, Parbatipur, Bangladesh; s University of Virginia, Charlottesville, VA, USA; t Instituto de Nutrición de Centroamérica y Panamá, Guatemala City, Guatemala; u University of Colorado-Anschutz Medical Campus, Denver, CO, USA; v Eunice Kennedy Shriver National Institute of Child Health and Human Development, National Institutes of Health, Bethesda, MD, USA

## Abstract

**Background:**

Sepsis is one of the leading causes of maternal mortality globally. In 2023, the Azithromycin Prevention in Labor Use (A-PLUS) trial showed intrapartum azithromycin for women planning a vaginal birth reduced the risk of maternal sepsis or death and infection. We aimed to evaluate the cost-effectiveness of intrapartum azithromycin for pregnant people planning a vaginal birth in low-income and middle-income countries (LMICs) using A-PLUS trial data.

**Methods:**

We compared the benefits and costs of intrapartum azithromycin versus standard care across 100 000 model simulations using data from the A-PLUS trial and a probabilistic decision tree model that included 24 mutually exclusive scenarios. A-PLUS was a randomised, double-blind, placebo-controlled trial that enrolled 29 278 women in labour at 28 weeks' gestation or more at eight sites in the Democratic Republic of the Congo, Kenya, Zambia, Bangladesh, India, Pakistan, and Guatemala. Women randomly assigned to azithromycin received a single intrapartum 2 g oral dose. In this cost-effectiveness analysis, we considered the cost of azithromycin treatment and its effects on a composite outcome of maternal infection, sepsis, or death and its individual components, and health-care use. Our analysis had a health-care sector perspective. We summarised results as an average and 95% CI of the model simulations. We also conducted sensitivity analyses. A-PLUS was registered at ClinicalTrials.gov, number NCT03871491.

**Findings:**

In model simulations, intrapartum azithromycin resulted in 1592·0 (95% CI 1139·7 to 2024·1) cases of maternal infection, sepsis, or death averted per 100 000 pregnancies, yielding 248·5 (95·3 to 403·7) facility readmissions averted, 866·8 (537·8 to 1193·2) unplanned clinic visits averted, and 1816·2 (1324·5 to 2299·7) antibiotic regimens averted. Using mean health-care costs across the A-PLUS sites, intrapartum azithromycin resulted in net savings of US$32 661 (–52 218 to 118 210) per 100 000 pregnancies and 13·2 (8·3 to 17·9) disability-adjusted life-years averted. The cost of facility readmission, cost of azithromycin, and probability of infection had the greatest impact on the incremental cost.

**Interpretation:**

In most cases, intrapartum azithromycin is a cost-saving intervention for the prevention of maternal infection, sepsis, or death in LMICs. This evidence supports global consideration of intrapartum azithromycin as an economically efficient preventive therapy to reduce infection, sepsis, or death among women planning a vaginal birth in LMICs.

**Funding:**

Eunice Kennedy Shriver National Institute of Child Health and Human Development and the Foundation for the National Institutes of Health through the Maternal, Newborn, and Child Health Discovery and Tools Initiative of the Bill & Melinda Gates Foundation

**Translations:**

For the French and Spanish translations of the abstract see Supplementary Materials section.

## Introduction

Globally, one woman dies in labour every 2 minutes.^1^ More than 95% of all maternal deaths occur in low-income and middle-income countries (LMICs), and sepsis is one of the major preventable causes of maternal death.^2^ Despite a focus on reducing maternal death in the UN Sustainable Development Goals, maternal mortality rates have continued to rise.^1^, ^3^ To reduce maternal mortality, there is an urgent, unmet need for affordable, effective, and safe therapies that can be implemented in LMICs.

In 2020, WHO's Global Maternal Sepsis Study (GLOSS) underlined the importance of obstetric and non-obstetric infections as contributors to maternal mortality.^4^ This evidence calls for effective strategies to manage maternal infection and sepsis.^5^ In the Azithromycin Prevention in Labor Use (A-PLUS; NCT03871491) trial, the National Institute for Child Health and Human Development (NICHD) Global Network for Women's and Children's Health Research identified a single dose of intrapartum azithromycin as effective in reducing maternal sepsis or death and specific maternal infections among women planning a vaginal birth in LMICs.^6^, ^7^ This large, multi-country randomised controlled trial showed a lower incidence of maternal, but not neonatal, sepsis or death among women who received 2 g of oral azithromycin in the intrapartum period compared with those who received placebo.^6^ A secondary analysis of the A-PLUS trial also indicated intrapartum azithromycin lowered the rate of maternal infections.^8^ In 2024, two systematic reviews and meta-analyses, which included the A-PLUS trial, reported findings consistent with the trial with regards to reductions in maternal sepsis and infection.^9^, ^10^

**Research in context**

**Evidence before this study**

In 2023, the A-PLUS trial conducted by the National Institute of Child Health and Human Development Global Network for Women's and Children's Health Research showed the efficacy of intrapartum azithromycin to prevent maternal infection, sepsis, or death in low-income and middle-income countries (LMICs). We searched PubMed for manuscripts on the cost-effectiveness of intrapartum azithromycin for improved maternal or neonatal outcomes from database inception to April 8, 2024, without language restriction using these search terms: “azithromycin” AND “cost-effectiveness” AND (“labor” OR “intrapartum” OR “pregnancy”) AND (“infection” OR “sepsis” OR “maternal mortality” OR “neonatal mortality”). Our search yielded 18 articles, of which two were cost-effectiveness analyses of azithromycin. Both studies evaluated the cost-effectiveness of azithromycin as adjunctive prophylaxis for caesarean delivery in high-income settings and had a moderate risk of bias.

**Added value of this study**

Our study shows the cost-effectiveness of intrapartum azithromycin for the prevention of maternal infection, sepsis, or death in women planning a vaginal birth in LMICs. Our analysis explored the cost-effectiveness of intrapartum azithromycin in this population in all countries combined, by region, and by individual country employing country-specific costs. In the analysis of all countries combined, intrapartum azithromycin resulted in a net savings of US$0·33 per parturient in LMICs, which translates to spending $0·91 per woman planning a vaginal birth to get back $1·24 per parturient. Although local conditions such as the cost of intrapartum azithromycin or the cost of a facility admission can differ, the intervention was cost saving or cost-effective in most countries analysed.

**Implications of all the available evidence**

This evidence supports global consideration of intrapartum azithromycin as an economically efficient preventative therapy to reduce infection, sepsis, or death among women planning a vaginal birth in LMICs.

To support policy makers considering which interventions provide the greatest value in maternal and child health, we sought to find out the cost-effectiveness of intrapartum azithromycin to prevent maternal infection, sepsis, or death. We aimed to estimate the incremental cost of intrapartum azithromycin per 100 000 pregnancies and the cost per case of maternal infection, sepsis, or death averted and per disability-adjusted life-year (DALY) averted after accounting for cost savings from reduced health-care use.

## Methods

### Study design and participants

In this study, we used a probabilistic decision tree model and data from the A-PLUS trial to estimate the cost-effectiveness of azithromycin to prevent maternal infection, sepsis, or death. A-PLUS evaluated the use of azithromycin for people in labour to prevent maternal or neonatal sepsis or death as a phase 3 randomised, double-blind, placebo-controlled trial.^6^ The trial enrolled participants from Sept 9, 2020, to Aug 18, 2022, at eight sites located in seven countries (the Democratic Republic of the Congo, Kenya, Zambia, Bangladesh, India [2 sites], Pakistan, and Guatemala). Women randomly assigned to the intervention received a single 2 g oral dose of azithromycin during labour. The primary maternal outcome was a composite of maternal sepsis or death. The study methods and results have been previously reported.^6^ Ethics committees and regulatory agencies at each of the eight participating sites, along with ethics committees of the US-based collaborators and the data coordinating centre at RTI International approved the A-PLUS trial protocol. All participants provided informed consent before enrolling in the trial (appendix 3 p 1). A-PLUS was registered at ClinicalTrials.gov, number NCT03871491.

Our cost-effectiveness analysis reflects a health-care sector perspective, accounting for only direct medical costs, without consideration for additional societal costs, such as lost wages. We chose this focus due to the absence of data in LMICs to inform a rigorous analysis from a broader societal perspective.

### Procedures

**Figure 1:**
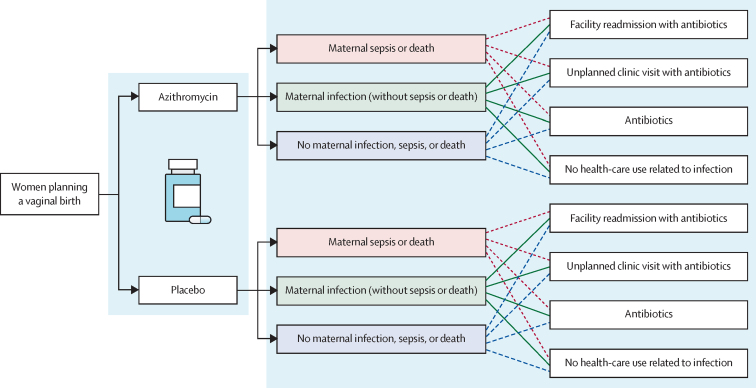
Model design

We developed a probabilistic decision tree model to compare maternal infection-related outcomes and associated health-care use between an azithromycin-treated group and a group receiving standard care (TreeAge Pro 2019, version 2.1). The model included 24 mutually exclusive scenarios (figure 1). We used this decision tree to compare costs and benefits between standard care and intrapartum azithromycin prophylaxis. The first branch of the model separated maternal outcomes into mutually exclusive branches of (1) maternal sepsis or death, (2) maternal infection (referring to cases of infection without associated sepsis or death), or (3) no maternal infection, sepsis, or death. The second branching of the model accounted for health-care use, with mutually exclusive categories of (1) readmission with antibiotics, (2) unplanned clinic visit with antibiotics, (3) antibiotics (without an unplanned clinic visit or readmission), or (4) no health-care use related to infection. No health-care use related to infection included unplanned clinic visits and readmissions that were not accompanied by the receipt of antibiotics. If a participant had both a readmission and an unplanned clinic visit, their health-care use was categorised as a readmission. All occurrences of antibiotics in the second branching of the model were for treatment, not prophylaxis.

We calculated the cost of azithromycin based on four 500 mg tablets and the median cost of US$0·91 per 2 g dosage from seven suppliers in the 2015 International Medical Products Price Guide (table 1).^11^ Data from a 2018 Health Action International report showed that costs for azithromycin in Kenya had decreased for the same purchaser over the period 2015–18; thus, we conservatively assumed that prices remained flat since 2015.^13^ Notably, we identified cost data since 2018 from one supplier (UNICEF) indicating slightly lower costs of a 2 g dosage with 250 mg tablets ($0·72) or substantially higher costs of a 2 g dosage with 500 mg tablets ($3·13) compared with the 2015 International Medical Products Price Guide estimate of $0·91 using 500 mg tablets.^14^ However, we determined that a median of seven suppliers from 2015 (ie, using the International Medical Products Price Guide cost of $0·91) was more representative than any single, more recent source given our assumption of flat pricing in the past 10 years.

**Table 1:** Parameters for all cost-effectiveness analyses

	**Placebo**	**Azithromycin**	**Distribution**	**Source**
**Treatment costs**				
Regimen cost	US$0·00	US$0·91*	Uniform	2015 International Medical Products Price Guide^11^
**Treatment effects**				
Relative risk of sepsis or death	1 (ref)	0·67	Log-normal	Tita et al (2023)^6^
Relative risk of infection (without sepsis or death)	1 (ref)	0·75	Log-normal	A-PLUS trial data
**Maternal outcome probabilities**				
Sepsis or death	0·023	0·015	Beta	A-PLUS trial data
Infection (without sepsis or death)	0·033	0·025	Beta	A-PLUS trial data
No infection, sepsis, or death	0·944	0·960	Calculated as remainder	A-PLUS trial data
**Health-care use probabilities of sepsis or death**				
Facility readmission with antibiotics	0·088	0·080	Beta	A-PLUS trial data
Unplanned clinic visit with antibiotics	0·326	0·237	Beta	A-PLUS trial data
Antibiotics	0·152	0·085	Beta	A-PLUS trial data
No health-care use related to infection	0·434	0·598	Beta	A-PLUS trial data
**Health-care use probabilities of infection (without sepsis or death)**				
Facility readmission with antibiotics	0·099	0·078	Beta	A-PLUS trial data
Unplanned clinic visit with antibiotics	0·474	0·449	Beta	A-PLUS trial data
Antibiotics	0·190	0·191	Beta	A-PLUS trial data
No health-care use related to infection	0·237	0·282	Beta	A-PLUS trial data
**Health-care use probabilities of no infection, sepsis, or death**				
Facility readmission with antibiotics	0·001	0·000	Beta	A-PLUS trial data
Unplanned clinic visit with antibiotics	0·004	0·004	Beta	A-PLUS trial data
Antibiotics	0·007	0·003	Beta	A-PLUS trial data
No health-care use related to infection	0·988	0·993	Beta	A-PLUS trial data
**Additional use parameters**				
Average length of stay for facility readmission	7·5	7·5	Normal	A-PLUS trial data
Average total stays if at least one readmission	1·2	1·2	Normal	A-PLUS trial data
Average number of clinic visits accompanying a readmission	0·1	0·1	Normal	A-PLUS trial data
Average total number of unplanned clinic visits if any unplanned clinic visits	1·1	1·1	Normal	A-PLUS trial data
**Disability weight**				
Maternal sepsis†	0·0169	0·0169	Point estimate	Ock et al (2016)^12^

* Cost of azithromycin regimen was based on four tablets of 500 mg with the median cost of US$0·2267 per tablet from the 2015 International Medical Products Price Guide.

† Disability weight for maternal sepsis (0·825)^12^ was annualised based on a duration of 7·5 days (average length of hospital stay for readmissions).

To estimate the treatment effect of azithromycin on maternal sepsis or death, we used previously published trial data.^6^ Based on a secondary analysis of the A-PLUS trial showing intrapartum azithromycin reduced maternal infection, we included the treatment effect on infection only (ie, excluding cases of sepsis or death) derived from the A-PLUS trial dataset.^8^ We also used the A-PLUS trial dataset to estimate baseline maternal outcome probabilities in the placebo group and adjusted probabilities for the treatment effect in the azithromycin-treated sample. To support analyses by region and by individual country, we estimated region-specific treatment effects and outcome probabilities (appendix 3 pp 2–3). This approach was supported by the original A-PLUS trial, which was powered to detect regional differences.^6^

We estimated health-care use probabilities separately for placebo and azithromycin groups to model additional benefits for health-care use supported by the primary manuscript using data from the trial.^6^ We categorised unplanned clinic visits and readmissions that were not accompanied by antibiotics for treatment in the group of no health-care use related to infection. We estimated the average length of stay for readmissions and the average number of unplanned clinic visits across the entire cohort. To support analyses by region and by individual country, we estimated region-specific health-care use probabilities and additional use parameters (appendix 3 pp 2–3).

We estimated an overall disability weight for maternal sepsis of 0·0169 using a previously reported disability weight of 0·85, which we annualised based on the average length of stay of 7·5 days.^12^ Although we considered using the Institute for Health Metrics and Evaluation Global Burden of Diseases, Injuries, and Risk Factors Study (IHME GBD) estimate of 0·133, this value seemed unrealistic for sepsis hospitalisation.^15^ The IHME GBD approach might not be well suited to this particular outcome of sepsis or their lay descriptions for the disability weight survey might not have captured the effect of severe infection (ie, high fever, pain, and feeling weak, which causes great difficulty with daily activities). Surprisingly, low disability weight estimates for other diseases in the IHME GBD, such as blindness, have also been noted.^16^ The disability weight from Ock and colleagues (0·85) was estimated from a large survey of physicians and medical students.^16^ Their assessment of the effect of maternal sepsis is based on more knowledge and experience than a member of the general population reading a brief lay description of a severe infection, which is used for GBD estimates.^12^ Notably, we did not find sufficient evidence in the published literature on the incidence of antimicrobial resistance (AMR) and its associated clinical risks following intrapartum azithromycin administration, nor its effect on cost (specifically, health-care use) to support the inclusion of AMR in this cost-effectiveness analysis.

For sensitivity analyses, we based low and high azithromycin cost estimates on the low ($0·60 per 2 g regimen) and high ($1·48 per 2 g regimen) price of azithromycin from the 2015 International Medical Products Price Guide.^11^ We derived the low and high estimates of azithromycin effectiveness on maternal sepsis or death and on maternal infection from the upper and lower 95% CIs published in the A-PLUS trial.^6^ Length of stay estimates were based on the IQR in the A-PLUS trial data (unpublished data). Health-care use costs were based on the range of costs from all study sites. Finally, probability ranges were based on ranges observed across the regions (Africa and Asia).

### Outcomes

The primary outcome of the A-PLUS trial was maternal sepsis or death within 6 weeks after delivery, with sepsis defined per WHO criteria as suspected or confirmed infection including fever or hypothermia with one or more signs of organ dysfunction. Maternal infection was a secondary outcome, defined as chorioamnionitis, endometritis, wound infections, abdominal or pelvic abscess, mastitis or breast abscess, pneumonia, or pyelonephritis. Our model presented individual and combined outcomes averted by azithromycin treatment, comprising cases of infection, sepsis, or death, cases of sepsis or death, and cases of infection (without sepsis or death). We expressed each outcome as cost-effectiveness results by calculating a cost per each type of case averted. To determine DALYs averted, we only considered years of life with disability (from hospital stays for sepsis). Our DALY estimates did not consider changes in mortality, as the A-PLUS trial did not show differences in mortality.

### Statistical analysis and cost-effectiveness

We analysed cost-effectiveness overall, by region (ie, Africa *vs* Asia), and by country. For the overall cost-effectiveness analysis, we used the estimates of key parameters reflecting overall A-PLUS trial data (table 1). For the regional analyses, we used estimates of key parameters reflecting region-specific data from the same trial dataset (appendix 3 pp 2–3). Country-specific analyses were based on region-specific estimates (ie, Africa *vs* Asia) except for the analysis for Guatemala, which was based on overall estimates. We used the average of all country-specific costs for the overall analysis, and the average of all African versus Asian country costs for regional analyses (appendix 3 p 4).

We assessed the average incremental cost-effectiveness across 100 000 model simulations comparing a model sample of women given azithromycin to a model sample of women receiving standard care.^6^ We evaluated cost-effectiveness using a ratio of cost per DALYs averted to gross domestic product (GDP) per capita, and determined thresholds for cost-effectiveness based on publications published in the past 5 years that considered interpretation of this ratio across various contexts.^17^, ^18^ Kazibwe and colleagues suggest a ratio of one to three times GDP is too arbitrary and probably not cost-effective in many countries. Because of insufficient resources and the high opportunity cost of forgoing other public health priorities, Kazibwe and colleagues recommended using locally developed cost-effectiveness thresholds if possible.^17^ In practice, thresholds are not available for many LMICs. A framework proposed in 2023 by Pinchon-Reviere and colleagues derives country-specific thresholds that incorporate local GDP, health spending, and life expectancy.^18^ Using their framework, 96% of low-income countries and 76% of lower-middle-income countries had a suggested threshold less than 0·5 GDP. The remaining countries had suggested thresholds between 0·5 and 1·0. Thus, we considered the intervention cost-effective if the ratio was less than 0·5 times GDP and moderately cost-effective if it was between 0·5 and 1·0 times GDP.

### Role of the funding source

A staff member from the Eunice Kennedy Shriver NICHD participated in interpreting data and reviewing and approving the manuscript. The funders had no further role in the following: design and conduct of the study; collection, management, analysis, and interpretation of the data; preparation, review, or approval of the manuscript; or decision to submit the manuscript for publication.

## Results

As previously reported,^6^ the A-PLUS trial enrolled 29 278 women who were randomly assigned to receive intrapartum azithromycin or placebo. Baseline characteristics, including maternal age, education, and parity, were similar between groups; delivery characteristics, such as induced labour and high risk for sepsis, were also similar between groups.^6^ Intrapartum azithromycin reduced maternal sepsis or death with a relative risk of 0·67 (95% CI 0·56–0·79; risk difference –0·8)^6^ and reduced maternal infection with a relative risk of 0·75 (95% CI 0·66–0·86; risk difference –1·6).^8^

In this cost-effectiveness analysis, overall results showed intrapartum azithromycin was associated with 1592·0 (95% CI 1139·7–2024·1) cases of infection, sepsis, or death averted per 100 000 pregnancies (table 2). These health outcomes resulted in 248·5 (95% CI 95·3–403·7) facility readmissions averted, 866·8 (537·8–1193·2) unplanned clinic visits averted, and 1816·2 (1324·5–2299·7) antibiotic regimens averted per 100 000 pregnancies (table 2). These results are broadly consistent with those reported in the A-PLUS trial.

**Table 2:** Cost-effectiveness of intrapartum azithromycin overall, in Africa, and in Asia

	**Overall**	**Africa**	**Asia**
**Health outcomes per 100 000 pregnancies**			
Cases of infection, sepsis, or death averted	1592·0 (1139·7 to 2024·1)	1996·4 (1747·6 to 2260·5)	1398·1 (637·5 to 2105·2)
Cases of sepsis or death averted	780·6 (486·9 to 1054·0)*	1632·5 (1404·7 to 1876·1)	248·8 (−193·2 to 631·1)
Cases of infection (without sepsis or death) averted	811·4 (460·7 to 1136·2)	363·9 (267·4 to 475·5)	1149·3 (516·0 to 1727·6)
Facility readmissions with antibiotics averted	248·5 (95·3 to 403·7)	208·4 (40·3 to 393·4)	302·2 (53·1 to 552·4)
Unplanned clinic visits with antibiotics averted	866·8 (537·8 to 1193·2)	956·9 (610·6 to 1320·9)	839·8 (336·5 to 1331·1)
Antibiotics only averted	1816·2 (1324·5 to 2299·7)	1528·8 (1061·3 to 2020·5)	2157·3 (1379·4 to 2919·7)
Disability-adjusted life-years averted	13·2 (8·3 to 17·9)	27·7 (23·8 to 31·8)	4·2 (−3·3 to 10·7)
**Cost-effectiveness outcomes, US$**			
Intervention cost† per 100 000 pregnancies	$90 963 (35 887 to 146 100)	$90 948 (35 932 to 146 094)	$90 927 (35 885 to 146 106)
Incremental cost per 100 000 pregnancies	−$32 661 (−118 210 to 52 218)	$27 198 (−40 681 to 94 195)	−$44 307 (−152 560 to 64 060)
Cost per case of infection, sepsis, or death averted	Cost saving	$13·62 (−20·04 to 48·75)	Cost saving
Cost per case of sepsis or death averted	Cost saving	$16·66 (−24·49 to 60·03)	Cost saving
Cost per case of infection (without sepsis or death) averted	Cost saving	$74·73 (−114·42 to 279·88)	Cost saving
Cost per disability-adjusted life-year averted	Cost saving	$982·92 (−1444·74 to 3541·51)	Cost saving

Data are n (95% CI). Overall model results are based on the estimates in table 1 and the average of all country-specific costs. Africa model results are based on the estimates in appendix 3 (p 2) and the average of all African country costs. Asia model results are based on the estimates in appendix 3 (p 3) and the average of all Asian country costs. All results represent the average with 95% CI of 100 000 model simulations, each using a different parameter set (drawn from the same distributions). Negative dollar values represent a net savings. Costs are presented in US$.

* Deaths contributed to 4·4% of these cases overall (3% in the placebo group and 6·6% in the intervention group) per the A-PLUS trial publication.^6^

† Intervention costs not exactly equal to $0·91 per person due to stochastic variation across the 100 000 samples.

By region, more cases of sepsis or death were averted in Africa (1632·5 [95% CI 1404·7 to 1876·1]) than in Asia (248·8 [–193·2 to 631·1]). By contrast, fewer cases of infection were averted in Africa (363·9 [267·4 to 475·5]) than in Asia (1149·3 [516·0 to 1727·6]; table 2). By region, there were 208·4 (40·3 to 393·4) facility readmissions averted in Africa and 302·2 (53·1 to 552·4) facility readmissions averted in Asia (table 2).

Overall cost-effectiveness results show net savings of $32 661 (95% CI –52 218 to 118 210) per 100 000 pregnancies, with cost savings per case of infection, sepsis, or death averted and per DALY averted (table 2). This saving equates to $0·33 saved per person who received azithromycin. As shown by the incremental cost-effectiveness ratio scatterplot, 99% of simulations found a cost of less than $100 per case of sepsis, death or infection averted, and the average of 100 000 simulations found this intervention to be cost saving (appendix 3 p 6). Using an average GDP across all countries ($2338) and 13·2 DALYs averted, we calculated an overall cost-effectiveness threshold of $29 541 per DALY averted (1·0 times GDP). Across 100 000 simulations, 75% of model results were cost saving and an additional 17% were cost-effective (<$29 541 per DALY averted).

By region, the incremental cost was $27 198 (95% CI –40 681 to 94 195) in Africa compared with net savings of $44 307 (–64 060 to 152 560) in Asia per 100 000 pregnancies (table 2). Accordingly, in Africa, the cost per case of sepsis, death, or infection averted was $13·62 (–20·04 to 48·75) and the cost per DALY averted was $982·92 (–1444·74 to 3541·51). Using $1403 as the average GDP per capita across the African countries, the intervention was moderately cost-effective at 0·70 times GDP. The intervention was cost saving in Asia (table 2). These results equate to $0·27 spent per treated person in labour in Africa, and $0·44 saved per treated person in labour in Asia. In individual country analysis, the incremental cost ranged from a high of $30 196 (95% CI –38 863 to 98 252) per 100 000 women in Zambia to net savings of $165 350 (23 654 to 308 055) in Guatemala (table 3). Results were cost saving in India, Pakistan, and Guatemala, and cost-effective in Kenya; the results for Zambia and Bangladesh were borderline cost-effective and the result for the Democratic Republic of the Congo was not cost-effective (table 3).^17^ Using 2022 national births, we also estimated the health-care cost increase or cost savings from a national implementation of intrapartum azithromycin, ranging from a cost increase of $1 049 889 in the Democratic Republic of the Congo to a cost savings of $19 231 163 in India (table 3).

**Table 3:** Cost-effectiveness of intrapartum azithromycin by country

	**Democratic Republic of the Congo**	**Kenya**	**Zambia**	**Bangladesh**	**India**	**Pakistan**	**Guatemala**
Intervention cost per 100 000 pregnancies	$90 948 (35 932 to 146 094)	$90 948 (35 932 to 146 094)	$90 948 (35 932 to 146 094)	$90 927 (35 885 to 146 106)	$90 927 (35 885 to 146 106)	$90 927 (35 885 to 146 106)	$90 963 (35 887 to 146 100)
Incremental cost per 100 000 pregnancies	$24 777 (−41 841 to 90 723)	$26 635 (−41 412 to 93 797)	$30 196 (−38 863 to 98 252)	$7704 (−73 039 to 88 533)	−$83 408 (−220 071 to 52 844)	−$79 313 (−205 728 to 47 221)	−$165 350 (−308 055 to −23 654)
Cost per case of infection, sepsis, or death averted	$12·41 (−20·67 to 46·97)	$13·34 (−20·43 to 48·56)	$15·13 (−19·18 to 50·79)	$5·51 (−53·2 to 89·41)	Cost saving	Cost saving	Cost saving
Cost per case of sepsis or death averted	$15·18 (−25·17 to 57·91)	$16·32 (−24·93 to 59·76)	$18·50 (−23·37 to 62·53)	$30·97 (−1190·51 to 1294·55)	Cost saving	Cost saving	Cost saving
Cost per case of infection (without sepsis or death) averted	$68·08 (−117·80 to 270·05)	$73·19 (−116·62 to 278·73)	$82·97 (−109·55 to 291·55)	$6·70 (−65·95 to 108·10)	Cost saving	Cost saving	Cost saving
Cost per DALY averted	$895·04 (−1485·02 to 3416·45)	$962·58 (−1470·69 to 3525·89)	$1091·26 (−1378·55 to 3689·01)	$1826·86 (−70 236·37 to 76 374·42)	Cost saving	Cost saving	Cost saving
GDP per capita	$654	$2099	$1457	$2688	$2411	$1589	$5473
Ratio of cost per DALY averted to GDP per capita*	1·37	0·46	0·75	0·8	NA	NA	NA
National births in 2022†	4 237 354	1 503 360	694 807	2 987 615	23 056 737	6 474 370	373 431
Estimated health-care cost in 2022‡	$1 049 889·20	$400 419·94	$209 803·92	$230 165·86	−$19 231 163·20	−$5 135 017·08	−$617 468·16

Data are n (95% CI), unless otherwise stated. Model results for the Democratic Republic of the Congo, Kenya, and Zambia are based on the estimates for the Africa region in appendix 3 (p 2). Model results for Bangladesh, India, and Pakistan are based on the estimates for the Asia region in appendix 3 (p 3). Model results for Guatemala are based on the overall estimates in table 1. All results represent the average with 95% CI of 100 000 model simulations, each using a different parameter set (drawn from the same distributions). Country-specific analyses reflect health-care use costs from each country in appendix 3 (p 4). Negative dollar values represent a net savings. Costs are presented in US$. DALY=disability-adjusted life-year. GDP=gross domestic product. NA=not applicable.

* Ratios less than 0·5 are considered cost-effective; ratios between 0·5 and 1·5 are considered borderline cost-effective.

† UNICEF DataWarehouse.

‡ Estimates obtained by multiplying annual births by $0·33 saved per person in labour.

**Figure 2:**
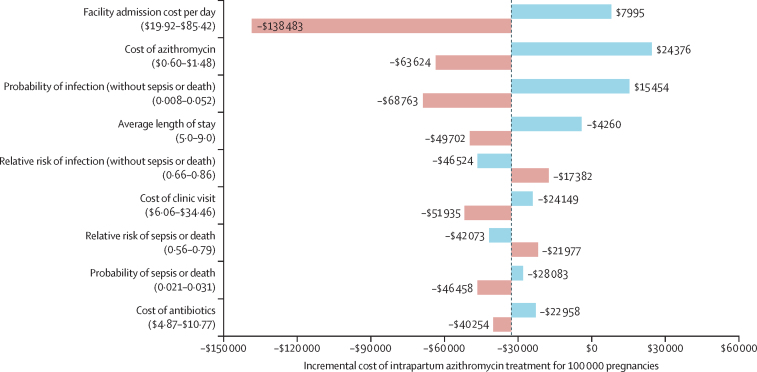
Sensitivity analyses of cost-effectiveness results of intrapartum azithromycin expressed per 100 000 pregnancies Horizontal bars are centred on the incremental cost in the base case (–$32 661). In each row, a key model parameter is varied across a range of feasible values, while all others are held constant at their point estimate from table 1 or table 2. Blue indicates the low parameter sensitivity input and red indicates the high parameter sensitivity input. Results are organised by their impact on the incremental cost of intrapartum azithromycin treatment. Costs are presented in US$.

One-way sensitivity analyses on azithromycin treatment cost yielded net savings of $63 624 (95% CI 55 254 to 70 681) in the low-cost scenario, compared with an incremental cost of $24 376 (–33 979 to 82 245) in the high-cost scenario, and a cost per DALY averted of $1842·27 (–2489·46 to 7506·69) in the high-cost scenario (appendix 3 p 5). One-way sensitivity analyses on the effectiveness of azithromycin in reducing sepsis or death and infection resulted in cost savings across all adverse health outcomes for both low and high effectiveness scenarios (appendix 3 p 5). Among key model parameters, the cost of facility readmission, the cost of azithromycin, and the probability of infection had the greatest impact on the incremental cost (figure 2).

## Discussion

Using data from the multi-country, large-scale A-PLUS trial,^6^ we found that with average LMIC health-care costs, intrapartum azithromycin for women planning a vaginal birth was a cost-saving intervention that reduced maternal infection, sepsis, or death. The cost per DALY averted in the overall analysis was cost saving.

By region, azithromycin was cost saving in Asia and borderline cost-effective in Africa. The higher incremental cost in Africa compared with net savings in Asia ($27 198 *vs* –$44 307; table 2) was driven by the lower costs of health-care use in Africa versus Asia, and particularly the lower cost of a facility readmission ($24·95 in Africa *vs* $34·18 in Asia; appendix 3 p 4); the lower number of infections averted and the lower number of facility readmissions in Africa compared with Asia also contributed. Despite the greater effectiveness of intrapartum azithromycin for infection in Africa than Asia (relative risk 0·56 *vs* 0·78; appendix 3 pp 2–3), the lower probability of infection in Africa than Asia (0·008 *vs* 0·052; appendix 3 pp 2–3) resulted in a lower number of infections averted in Africa compared with Asia (364 *vs* 1149; table 2). We observed a lower number of facility readmissions averted in Africa compared with Asia (208 *vs* 302; table 2) despite greater effectiveness of azithromycin on reducing readmissions in Africa for both sepsis or death and infection (appendix 3 pp 2–3). In summary, cost savings from reductions in health-care use exceeded the cost of the intervention in Asia, resulting in net savings. Although this was not the case in Africa, intrapartum azithromycin was still cost-effective, as evidenced by a cost per DALY averted to GDP per capita ratio of less than 1·0.

In individual country analyses, our results ranged from cost savings to not cost-effective (>1·0 times GDP per DALY averted). Although the cost per DALY averted in each of the African countries was similar, the variance in the GDP per capita resulted in cost-effective results in Kenya compared with borderline cost-effectiveness in Zambia and not cost-effective in the Democratic Republic of the Congo. Intrapartum azithromycin in India and Pakistan was cost saving; by contrast, the absence of cost savings in Bangladesh was driven by lower health-care costs compared with the other Asian countries.

Our cost-effectiveness results were particularly sensitive to the price of the azithromycin regimen. We based the price of azithromycin used in our model on two publications that estimated cost before the COVID-19 pandemic, which indicated a decrease in costs from 2015 to 2018 for the same purchaser.^13^, ^19^ Medication costs can shift based on demand, which could have increased for azithromycin after the COVID-19 pandemic. Our sensitivity analysis illustrates the effect of shifts in the cost of azithromycin on our results, with a high-cost scenario resulting in a cost per DALY averted of $1842·27 (appendix 3 p 5).

The cost savings of azithromycin use in the intrapartum period have been previously shown in the context of prophylactic use before caesarean delivery in a high-income setting. In 2016, a multicentre randomised trial in the USA (C/SOAP trial) showed adjunctive azithromycin prophylaxis for caesarean delivery reduced the risk of infection by 50%.^20^ The companion economic evaluation indicated that adjunctive azithromycin prophylaxis resulted in savings of $360 (95% CI 155–451) per caesarean delivery.^21^ These cost savings were corroborated by a theoretical decision analytical model.^22^ Similarly, our results also showed cost savings of intrapartum azithromycin in LMICs. However, in some scenarios with lower facility costs, higher azithromycin costs, or lower probabilities of infection, intrapartum azithromycin was cost-effective under the thresholds we used but not cost saving (figure 2). Given our base case cost of azithromycin ($0·91), the intervention is not considered cost-effective in the Democratic Republic of the Congo. However, at a lower cost of azithromycin, such as the $0·60 azithromycin cost used in our sensitivity analysis (appendix 3 p 5), the intervention would be cost saving in the Democratic Republic of the Congo.

Notably, our analysis reflects data from a clinical trial and might not reflect real-world conditions with considerations of compliance and various standards of health care. We expect only small reductions in medication compliance in real-world settings due to administration of the intervention during a facility admission and the one-time dose of the medication.^23^ The trial did not measure baseline differences in standard of care related to facility-based infection prevention measures, which vary substantially.^24^ Quality infection prevention and control is crucial for reducing sepsis and should be strongly recommended as an essential strategy to reduce maternal sepsis. As described in the primary analysis of the A-PLUS trial, the frequent use of prophylactic antibiotics, particularly in the non-African sites, probably blunted the effect of the intervention. A policy recommendation in support of intrapartum azithromycin might reduce the use of other prophylactic antibiotics, thus potentially increasing the cost benefits of intrapartum azithromycin when implemented routinely.

Our analysis was strengthened by the volume and specificity of data in the A-PLUS trial.^6^ We used published results from the A-PLUS trial to estimate treatment effect and probabilities of health outcomes. We used primary data from the A-PLUS trial to define probabilities for health-care use. We estimated the costs of health-care use in the same study sites as the trial through a survey, and we incorporated estimates of subsidies for better cross-site comparability.

Our results should be considered within the context of potential limitations. We derived our estimate of the disability weight for maternal sepsis from a single publication in one LMIC, which might not be generalisable to all LMICs. In the African countries where intrapartum azithromycin was borderline cost-effective, shifts in this estimate could affect results. Although our cost range for azithromycin represents our best estimate based on the data available to us, we understand that the cost could vary widely from region to region or under constrained supply conditions. The A-PLUS trial required confirmation of infection to classify the indication of antibiotics as treatment; hence, prophylactic antibiotic use was probably overestimated, and our results for health-care use averted and cost-effectiveness probably an underestimate. Our incremental cost estimates do not include the administrative and logistical costs of implementing a national programme for intrapartum azithromycin. In small countries, such as Guatemala or Zambia, these might be significant compared with the costs or cost savings. The A-PLUS trial did not include data on baseline differences in maternal nutritional status or inflammation, thus we cannot make direct assessments about the effects of intrapartum azithromycin compared with maternal nutritional interventions on sepsis. Notably, the A-PLUS trial found no effect of intrapartum azithromycin on neonatal sepsis or death; an alternative antibiotic for women who are in labour that reduces both maternal and neonatal morbidity and mortality might be preferable. Our analysis was limited to a health-care sector perspective, and did not consider the economic effect of a potential neuroprotective benefit of azithromycin for neonates.^25^

Although the results of the A-PLUS trial and this cost-effectiveness analysis were promising, real-world implementation of intrapartum azithromycin requires several additional considerations. Azithromycin carries a risk of QT interval prolongation and fatal arrhythmias per US Federal Drug Administration data.^26^ Given this risk, the A-PLUS trial excluded women with arrhythmias or known cardiomyopathy. Recommendations for large-scale implementation of azithromycin should include clinical algorithms that limit its use among women at high risk of side-effects (eg, those with underlying cardiac disease including proarrhythmic conditions or known prolongation of the QT interval, or those taking concurrent medications that prolong the QT interval). The potential effect of recommending large-scale administration of antibiotic prophylaxis with intrapartum azithromycin on both maternal and neonatal AMR and the microbiome should be noted. There are few published data on long-term adverse outcomes and AMR following such administration.^9^ Following intrapartum azithromycin administration in The Gambia and Burkina Faso, *Escherichia coli* carriage in infants decreased but *Klebsiella pneumoniae* carriage and azithromycin resistance in both bacteria increased.^27^ The NICHD Global Network for Women's and Children's Health Research is evaluating AMR and the microbiome as a sub-study of the A-PLUS trial, with both maternal and neonatal surveillance conducted up to 12 months postpartum.^28^ The results of this study will be particularly important to understand unintended effects of the intervention and their implications for real-world programmes. Future cost-effectiveness analyses of intrapartum azithromycin should be expanded to include the economic effect of AMR and microbiome effects.

In conclusion, we found intrapartum azithromycin results in net savings of $0·33 per person giving birth in LMICs; this result translates to spending $0·91 to get back $1·24 per woman planning a vaginal birth. Although local conditions, such as the cost of azithromycin or the cost of a facility admission might differ, the intervention was cost saving or cost-effective in most countries analysed. This evidence supports global consideration of intrapartum azithromycin as an economically efficient preventive therapy to reduce infection, sepsis, or death among women planning a vaginal birth in LMICs. There is a crucial need for further studies to delineate the implications of large-scale implementation of azithromycin prophylaxis on AMR and clinical risks; the results of such studies should be incorporated into future cost-effectiveness analyses. Future trials should also consider the relative efficacy and cost-effectiveness of intrapartum azithromycin to non-antibiotic interventions (eg, infection prevention and control, improving quality of intrapartum care, and health promotion strategies) aimed at reducing maternal sepsis.

### Contributors

### Equitable partnership declaration

The authors of this paper have submitted an equitable partnership declaration (appendix 4). This statement allows researchers to describe how their work engages with researchers, communities, and environments in the countries of study. This statement is part of *The Lancet Global Health*'s broader goal to decolonise global health.

### Data sharing

De-identified participant data from the A-PLUS trial is available at the National Institute for Child Health and Human Development data repository (N-DASH): https://dash.nichd.nih.gov/. Data sharing is accessed and governed according to the procedures and policies of N-DASH. A data dictionary is also provided.

## Declaration of interests

All authors other than MK-T received National Institute for Child Health and Human Development grant funding for this work; AT received additional funding from Mirvie, the American Heart Association, and Pfizer for research related to this work.

## Supplementary Materials

French translation of the abstract

Spanish translation of the abstract

Supplementary appendix 3

Equitable Partnership Declaration
