# Supplementary material for: Cost-effectiveness of intrapartum azithromycin to prevent maternal infection, sepsis, or death in low-income and middle-income countries: a modelling analysis of data from a randomised, multicentre, placebo-controlled trial
Source: Lancet Glob Health. 2025 Mar 26;13(4):e679–88. doi: 10.1016/S2214-109X(24)00517-5 (PMC11950424; doi:10.1016/S2214-109X(24)00517-5)
Supplement: French translation of the abstract [file mmc1.pdf]

THE LANCET
Global Health

Supplementary appendix 1

This translation in French was submitted by the authors and we reproduce it as supplied. It has not been peer reviewed. *The Lancet*'s editorial processes have only been applied to the original in English, which should serve as reference for this manuscript.

Cette traduction en français a été proposée par les auteurs et nous l'avons reproduite telle quelle. Elle n'a pas été examinée par des pairs. Les processus éditoriaux du *Lancet* n'ont été appliqués qu'à l'original en anglais et c'est cette version qui doit servir de référence pour ce manuscrit.

Supplement to: Patterson JK, Neuwahl S, Kirsch S, et al. Cost-effectiveness of intrapartum azithromycin to prevent maternal infection, sepsis, or death in low-income and middle-income countries: a modelling analysis of data from a randomised, multicentre, placebo-controlled trial. *Lancet Glob Health* 2025; **13:** e679–88.

Coût-efficacité de l'azithromycine intrapartum pour prévenir l'infection, le sepsis ou le décès maternels dans les pays à revenu faible ou intermédiaire : analyse de modélisation des données d'un essai randomisé, multicentrique et contrôlé par placebo

Résumé

Contexte : Le sepsis est l'une des principales causes de mortalité maternelle dans le monde. En 2023, l'essai A-PLUS (Azithromycin Prevention in Labor Use) a démontré que l'azithromycine intrapartum administrée aux femmes prévoyant un accouchement par voie vaginale réduisait le risque de sepsis ou de décès maternel et d'infection. Nous avons cherché à évaluer le rapport coût-efficacité de l'azithromycine intrapartum pour les femmes enceintes prévoyant un accouchement par voie vaginale dans les pays à faible revenu et à revenu intermédiaire (PRFI) en utilisant les données de l'essai A-PLUS.

Méthodes : Nous avons comparé les avantages et les coûts de l'azithromycine intrapartum par rapport aux soins standard sur 100 000 simulations de modèles en utilisant les données de l'essai A-PLUS et un modèle d'arbre de décision probabiliste qui comprenait 24 scénarios mutuellement exclusifs. L'essai A-PLUS est un essai randomisé, en double aveugle, contrôlé par placebo, auquel ont participé 29 278 femmes en travail à 28 semaines de gestation ou plus, sur huit sites en République démocratique du Congo, au Kenya, en Zambie, au Bangladesh, en Inde, au Pakistan et au Guatemala. Les femmes assignées de manière aléatoire à l'azithromycine ont reçu une dose unique de 2 g par voie orale au cours de l'accouchement. Dans cette analyse coût-efficacité, nous avons pris en compte le coût du traitement à l'azithromycine et ses effets sur une issue composite (infection maternelle, sepsis ou décès) et ses composantes individuelles, ainsi que sur l'utilisation des soins de santé. Notre analyse s'est inscrite dans la perspective du secteur des soins de santé. Nous avons résumé les résultats sous la forme d'une moyenne et d'un IC à 95 % des simulations du modèle. Nous avons également effectué des analyses de sensibilité. A-PLUS a été enregistré sur ClinicalTrials.gov, sous le numéro NCT03871491.

Résultats : Dans les simulations du modèle, l'azithromycine intra-partum a permis d'éviter 1592·0 (IC 95 % 1139·7 à 2024·1) cas d'infection, de sepsis ou de décès maternels pour 100 000 grossesses, ce qui a permis d'éviter 248·5 (95·3 à 403·7) réadmissions en établissement, 866·8 (537·8 à 1193·2) visites imprévues en clinique et 1816·2 (1324·5 à 2299·7) régimes d'antibiotiques. Sur la base des coûts moyens des soins de santé dans l'ensemble des sites A-PLUS, l'azithromycine intrapartum a permis une économie nette de $32 661 (-52 218 à 118 210) pour 100 000 grossesses et 13·2 (8·3 à 17·9) années de vie corrigées de l'incapacité évitées. Le coût de la réadmission en établissement, le coût de

l'azithromycine et la probabilité d'infection ont eu le plus grand impact sur le coût différentiel.

Interprétation : Dans la plupart des cas, l'azithromycine intrapartum est une intervention rentable pour la prévention de l'infection, de sepsis ou du décès maternels dans les PRFI. Ces données soutiennent la considération globale de l'azithromycine intrapartum comme une thérapie préventive économiquement efficace pour réduire l'infection, le sepsis ou le décès chez les femmes qui prévoient un accouchement par voie vaginale dans les PRFI.

Financement : Eunice Kennedy Shriver National Institute of Child Health and Human Development et Foundation for the National Institutes of Health par l'intermédiaire de la Maternal, Newborn, and Child Health Discovery and Tools Initiative de la Bill & Melinda Gates Foundation.
