# Supplementary material for: Cost-effectiveness of intrapartum azithromycin to prevent maternal infection, sepsis, or death in low-income and middle-income countries: a modelling analysis of data from a randomised, multicentre, placebo-controlled trial
Source: Lancet Glob Health. 2025 Mar 26;13(4):e679–88. doi: 10.1016/S2214-109X(24)00517-5 (PMC11950424; doi:10.1016/S2214-109X(24)00517-5)
Supplement: Spanish translation of the abstract [file mmc2.pdf]

THE LANCET
Global Health

Supplementary appendix 2

This translation in Spanish was submitted by the authors and we reproduce it as supplied. It has not been peer reviewed. *The Lancet*'s editorial processes have only been applied to the original in English, which should serve as reference for this manuscript.

Los autores nos proporcionaron esta traducción al español y la reproducimos tal como nos fue entregada. No la hemos revisado. Los procesos editoriales de *The Lancet* se han aplicado únicamente al original en inglés, que debe servir de referencia para este manuscrito.

Supplement to: Patterson JK, Neuwahl S, Kirsch S, et al. Cost-effectiveness of intrapartum azithromycin to prevent maternal infection, sepsis, or death in low-income and middle-income countries: a modelling analysis of data from a randomised, multicentre, placebo-controlled trial. *Lancet Glob Health* 2025; **13:** e679–88.

Coste-efectividad de la azitromicina intraparto para prevenir la infección materna, la sepsis o la muerte en países de ingresos bajos y medios: un análisis de modelización de los datos de un ensayo aleatorizado, multicéntrico y controlado con placebo.

Resumen

Antecedentes: La sepsis es una de las principales causas de mortalidad materna en todo el mundo. En 2023, el ensayo Uso Preventivo de Azitromicina durante el Parto (del inglés, Azithromycin Prevention in Labor Use, A-PLUS) mostró que la azitromicina intraparto para las mujeres que planificaban un parto vaginal reducía el riesgo de sepsis materna o muerte e infección. El objetivo fue evaluar la relación coste-efectividad de la azitromicina intraparto para las embarazadas que planifican un parto vaginal en países de ingresos bajos y medios (PRMB) utilizando los datos del ensayo A-PLUS.

Métodos: Se compararon los beneficios y costes de la azitromicina intraparto frente a la atención estándar a través de 100 000 simulaciones de modelos utilizando datos del ensayo A-PLUS y un modelo de árbol de decisión probabilístico que incluía 24 escenarios mutuamente excluyentes. A-PLUS fue un ensayo aleatorizado, doble ciego y controlado con placebo en el que participaron 29 278 parturientas con 28 semanas de gestación o más en ocho centros de la República Democrática del Congo, Kenia, Zambia, Bangladesh, India, Pakistán y Guatemala. Las mujeres asignadas aleatoriamente a azitromicina recibieron una única dosis oral intraparto de 2 g. En este análisis de coste-efectividad, consideramos el coste del tratamiento con azitromicina y sus efectos sobre un resultado compuesto de infección materna, sepsis o muerte y sus componentes individuales, y el uso de asistencia sanitaria. Nuestro análisis tenía una perspectiva del sector sanitario. Resumimos los resultados como media e IC del 95% de las simulaciones del modelo. También se realizaron análisis de sensibilidad. A-PLUS se registró en ClinicalTrials.gov, con el número NCT03871491.

Resultados: En las simulaciones del modelo, la azitromicina intraparto evitó 1592·0 (IC 95%: 1139·7 a 2024·1) casos de infección, sepsis o muerte materna por cada 100 000 embarazos, lo que produjo 248·5 (95·3 a 403·7) readmisiones evitadas, 866·8 (537·8 a 1193·2) visitas clínicas no planificadas y 1816·2 (1324·5 a 2299·7) regímenes antibióticos evitados. Utilizando los costes sanitarios medios de los centros A-PLUS, la azitromicina intraparto produjo un ahorro neto de USD $32 661 (-52 218 a 118 210) por 100 000 embarazos y evitó 13·2 (8·3 a 17·9) años de vida ajustados en función de la discapacidad. El coste del reingreso en el centro, el coste de la azitromicina y la probabilidad de infección fueron los factores que más influyeron en el coste incremental.

Interpretación: En la mayoría de los casos, la azitromicina intraparto es una intervención que ahorra costos para la prevención de la infección materna, la sepsis o la muerte en los PBI y los PIM. Esta evidencia apoya la consideración global de la azitromicina intraparto como una terapia preventiva económicamente eficiente para reducir la infección, la sepsis o la muerte entre las mujeres que planean un parto vaginal en los PIBM.

Financiación: Eunice Kennedy Shriver National Institute of Child Health and Human Development, USA y Foundation for the National Institutes of Health through the Maternal, Newborn, and Child Health Discovery and Tools Initiative of The Bill & Melinda Gates Foundation, USA.
