# Supplementary material for: Cost-effectiveness of intrapartum azithromycin to prevent maternal infection, sepsis, or death in low-income and middle-income countries: a modelling analysis of data from a randomised, multicentre, placebo-controlled trial
Source: Lancet Glob Health. 2025 Mar 26;13(4):e679–88. doi: 10.1016/S2214-109X(24)00517-5 (PMC11950424; doi:10.1016/S2214-109X(24)00517-5)

THE LANCET
Global Health

Supplementary appendix 3

This appendix formed part of the original submission and has been peer reviewed. We post it as supplied by the authors.

Supplement to: Patterson JK, Neuwahl S, Kirsch S, et al. Cost-effectiveness of intrapartum azithromycin to prevent maternal infection, sepsis, or death in low-income and middle-income countries: a modelling analysis of data from a randomised, multicentre, placebo-controlled trial. *Lancet Glob Health* 2025; **13:** e679–88.

Supplemental Table 1: **Institutional Regulatory Board Numbers for the A-PLUS Trial**

**Name and Location of Implementing Site**		**PI/IPI**	**Site FWA #**
**GN03-Zambia**	*University of Alabama, Birmingham*	Waldemar Carlo (PI)	FWA00005960
	University of Zambia, Lusaka, Zambia	Elwyn Chomba (IPI)	FWA00000338
**GN02-DRC**	*University of North Carolina School of Medicine, Chapel Hill, NC*	Melissa Bauserman (PI)	FWA00004801
	Kinshasa School of Public Health, Kinshasa, Democratic Republic of Congo (DRC)	Antoinette Tshefu (IPI)	FWA00003581
**GN06-Guatemala**	*University of Colorado Denver Anschutz Medical Campus*	Nancy Krebs (PI)	FWA00005070
	Institute for Nutrition in Central America and Panama (INCAP), Guatemala City, Guatemala	Manolo Mazariegos (IPI)	FWA00000742
**GN07-Bangladesh**	*University of Virginia*	William Petri (PI)	FWA00006183
	The International Center for Diarrheal Disease Research, Dhaka, Bangladesh	Rashidul Haque (IPI)	FWA00001468
**GN08- Belagavi, India**	*Thomas Jefferson University*	Richard Derman (PI)	FWA00002109
	JN Medical College, Belgaum, India	Shivaprasad Goudar (IP)	FWA00000766
**GN09- Pakistan**	*Columbia University*	Robert Goldenberg (PI)	FWA00003831
	Aga Khan University, Karachi, Pakistan	Sarah Saleem (IPI)	FWA00001177
**GN11-<br>Nagpur, India**	*Boston University*	Patricia Hibberd (PI)	FWA00003136
	Lata Medical Research Foundation, Nagpur, India	Archana Patel (IPI)	FWA00012971
**GN12-Kenya**	*Indiana University School of Medicine, Indianapolis, Indiana*	Edward Liechty (PI)	FWA00003544
	Moi University School of Medicine, Eldoret, Kenya	Fabian Esamai (IPI)	FWA00003128

***Supplemental Table 2:* Parameters for all cost-effectiveness analyses for Africa**

	Placebo	Azithromycin	Distribution	Source
**Treatment costs**	··	··	··	··
Regimen cost [a]	US$0·00	US$0·91	Uniform	2015 International Medical Products Price Guide[11]
**Treatment effects**	··	··	··	··
Relative risk of sepsis or death	1 (ref)	0·47	Log-normal	Tita, 2023[7]
Relative risk of infection (without sepsis or death)	1 (ref)	0·56	Log-normal	A-PLUS trial data
**Maternal outcome probabilities**	··	··	··	··
Sepsis or death	0·031	0·015	Beta	A-PLUS trial data
Infection (without sepsis or death)	0·008	0·005	Beta	A-PLUS trial data
No infection, sepsis, or death	0·961	0·980	Calculated as remainder	A-PLUS trial data
**Healthcare use probabilities for sepsis or death**	··	··	··	A-PLUS trial data
Facility readmission with antibiotics	0·096	0·060	Beta	A-PLUS trial data
Unplanned clinic visit with antibiotics	0·508	0·506	Beta	A-PLUS trial data
Antibiotics	0·079	0·084	Beta	A-PLUS trial data
No healthcare use related to infection	0·317	0·350	Beta	A-PLUS trial data
**Healthcare use probabilities for infection (without sepsis or death)**	··	··	··	A-PLUS trial data
Facility readmission with antibiotics	0·021	0·074	Beta	A-PLUS trial data
Unplanned clinic visit with antibiotics	0·583	0·667	Beta	A-PLUS trial data
Antibiotics	0·167	0·111	Beta	A-PLUS trial data
No healthcare use related to infection	0·229	0·148	Beta	A-PLUS trial data
**Healthcare use probabilities for no infection, sepsis, or death**	··	··	··	A-PLUS trial data
Facility readmission with antibiotics	0·000	0·000	Beta	A-PLUS trial data
Unplanned clinic visit with antibiotics	0·001	0·001	Beta	A-PLUS trial data
Antibiotics	0·003	0·001	Beta	A-PLUS trial data
No healthcare use related to infection	0·996	0·998	Beta	A-PLUS trial data
**Additional use parameters**	··	··	··	A-PLUS trial data
Average length of stay for facility readmission	5·8	5·8	Normal	A-PLUS trial data
Average total stays if at least one readmission	1·1	1·1	Normal	A-PLUS trial data
Average number of clinic visits accompanying a readmission	0·1	0·1	Normal	A-PLUS trial data
Average total number of unplanned clinic visits if any unplanned clinic visits	1·1	1·1	Normal	A-PLUS trial data
**Disability weight**	··	··	··	··
Maternal sepsis [b]	0·0169	0·0169	Point estimate	Ock, 2016[14]

[a] Cost of azithromycin regimen was based on four tablets of 500 mg using the median cost of US$0·2267 per tablet from the 2015 International Medical Products Price Guide.

[b] Disability weight for maternal sepsis (0·825[14]) was annualized based on a duration of 7·5 days (the overall average length of hospital stay for readmissions). For regional analyses, we used the same overall disability weight to avoid assigning different disability weights for the same outcome.

Supplemental Table 3: **Parameters for all cost-effectiveness analyses for Asia**

	Placebo	Azithromycin	Distribution	Source
**Treatment costs**	··	··	··	··
Regimen cost [a]	US$0·00	US$0·91	Uniform	2015 International Medical Products Price Guide[11]
**Treatment effects**	··	··	··	··
Relative risk of sepsis or death	1 (ref)	0·88	Log-normal	Tita, 2023[7]
Relative risk of infection (without sepsis or death)	1 (ref)	0.78	Log-normal	A-PLUS trial data
**Maternal outcome probabilities**	··	··		··
Sepsis or death	0·021	0·019	Beta	A-PLUS trial data
Infection (without sepsis or death)	0·052	0·041	Beta	A-PLUS trial data
No infection, sepsis, or death	0·928	0.940	Calculated as remainder	A-PLUS trial data
**Healthcare use probabilities for sepsis or death**	··	··		A-PLUS trial data
Facility readmission with antibiotics	0·080	0·093	Beta	A-PLUS trial data
Unplanned clinic visit with antibiotics	0·123	0·071	Beta	A-PLUS trial data
Antibiotics only	0·233	0·086	Beta	A-PLUS trial data
No healthcare use related to infection	0·564	0·750	Beta	A-PLUS trial data
**Healthcare use probabilities for infection (without sepsis or death)**	··	··	··	A-PLUS trial data
Facility readmission with antibiotics	0·112	0·081	Beta	A-PLUS trial data
Unplanned clinic visit with antibiotics	0·457	0·424	Beta	A-PLUS trial data
Antibiotics	0·196	0·202	Beta	A-PLUS trial data
No healthcare use related to infection	0·235	0·293	Beta	A-PLUS trial data
**Healthcare use probabilities for no infection, sepsis, or death**	··	··	··	A-PLUS trial data
Facility readmission with antibiotics	0·001	0·000	Beta	A-PLUS trial data
Unplanned clinic visit with antibiotics	0·007	0·006	Beta	A-PLUS trial data
Antibiotics	0·011	0·005	Beta	A-PLUS trial data
No healthcare use related to infection	0·981	0·989	Beta	A-PLUS trial data
**Additional use parameters**	··	··	··	A-PLUS trial data
Average length of stay for facility readmission	7·8	7·8	Normal	A-PLUS trial data
Average total stays if at least one readmission	1·2	1·2	Normal	A-PLUS trial data
Average number of clinic visits accompanying a readmission	0·2	0·2	Normal	A-PLUS trial data
Average total number of unplanned clinic visits if any unplanned clinic visits	1·1	1·1	Normal	A-PLUS trial data
**Disability weight**	··	··	··	··
Maternal sepsis [b]	0·0169	0·0169	Point estimate	Ock, 2016[14]

[a] Cost of azithromycin regimen was based on four tablets of 500 mg using the median cost of US$0·2267 per tablet from the 2015 International Medical Products Price Guide.

[b] Disability weight for maternal sepsis (0·825[14]) was annualized based on a duration of 7·5 days (the overall average length of hospital stay for readmissions). For regional analyses, we used the same overall disability weight to avoid assigning different disability weights for the same outcome.

Supplemental Table 4: **Cost parameters for cost-effectiveness analyses by country**

**Country**	**Overall Average**	**Africa Average**	**Asia Average**	**DRC**	**Kenya [a]**	**Zambia**	**Bangladesh [b]**	**India**	**Pakistan**	**Guatemala**
**Cost source**	N/A	N/A	N/A	Patient payment amounts	Patient payment amounts	Accounting cost	Patient payment amounts	Patient payment amounts	Accounting cost	Accounting cost
**Facility admission cost per day, USD [c]**	38·10	24·95	34·18	21·60	25·24	28·00	19·92	48·43	42·26	85·42
**Clinic visit cost, USD [d]**	14·76	10·80	12·16	10·20	11·56	10·64 [e]	6·06	13·62	16·06 [e]	34·46
**Antibiotic cost, USD**	8·18	7·43	8·06	10·20	7·22	4·87	6·34	7·26	10·58	10·77

[a] Estimates from the site included subsidized costs; we added the average cost per day of lab tests and radiology to account for this subsidy.

[b] Estimates from the site included subsidized costs; we doubled costs to represent the costs more typical of private healthcare in Bangladesh.

[c] The total cost of a facility readmission in our model reflects the cost of a facility admission considering the average length of stay with the additional cost of antibiotics, and accounting for the average total readmissions (Table 1 for overall analysis; Supplemental Tables 1 & 2 for regional analyses); we also accounted for the cost associated with the average number of clinic visits accompanying a readmission.

[d] The total cost of an unplanned clinic visit in our model reflects the cost of an unplanned clinic visit with the additional cost of antibiotics, and accounts for the average cost of the total number of clinic visits attended across the study period (Table 1 for overall analysis; Supplemental Tables 1 & 2 for regional analyses).

[e] Assumed the cost of a clinic visit was 38% of a facility admission based on the average ratio from other countries in the table with non-missing clinic visit data.
Abbreviations: DRC=Democratic Republic of the Congo, N/A=non-applicable, USD=United States dollars.

Supplemental Table 5: **One-way sensitivity analyses on the cost and effectiveness of intrapartum azithromycin**

**Azithromycin treatment cost** [a]			
	**Base case azithromycin cost (0·91)**	**Low azithromycin cost (0·60)**	**High azithromycin cost (1·48)**
**Parameters adjusted (95% CI)**	··	··	··
Intervention cost per 100,000 pregnancies, USD	90,963 [b] (35,887, 146,100)	60,000	148,000
**Cost-effectiveness outcomes, USD (95% CI)**	··	··	··
Incremental cost per 100,000 pregnancies	-32,661 (-118,210, 52,218)	-63,624 (-70,681, -55,254)	24,376 (-33,979, 82,245)
Cost per case of infection, sepsis, or death averted	Cost-saving	Cost-saving	15·31 (-19·95, 60·72)
Cost per case of sepsis or death averted	Cost-saving	Cost-saving	31·23 (-42·2, 127·24)
Cost per case of infection (without sepsis or death) averted	Cost-saving	Cost-saving	30·04 (-39·42, 133·89)
Cost per DALY averted	Cost-saving	Cost-saving	1,842·27 (-2,489·46, 7,506·69)
**Azithromycin effectiveness in reducing sepsis or death** [c]			
	**Base case azithromycin effectiveness (RR 0·67)**	**High azithromycin effectiveness (RR 0·56)**	**Low azithromycin effectiveness (RR 0·79)**
**Parameters adjusted (95% CI)**	··	··	··
Cases of sepsis or death averted	780·6 (486·9, 1054·0)	1033·63 (928·49, 1143·89)	493·33 (443·15, 545·95)
**Cost-effectiveness outcomes, USD (95% CI)**	··	··	··
YLDs averted per 100,000 pregnancies	13·2 (8·3, 17·9)	17·5 (15·7, 19·4)	8·4 (7·5, 9·3)
Incremental cost per 100,000 pregnancies	-32,661 (-118,210, 52,218)	-42,073 (-126,366, 41,159)	-21,977 (-107,955, 63,297)
Cost per case of infection, sepsis, or death averted	Cost-saving	Cost-saving	Cost-saving
Cost per case of sepsis or death averted	Cost-saving	Cost-saving	Cost-saving
Cost per case of infection (without sepsis or death) averted	Cost-saving	Cost-saving	Cost-saving
Cost per DALY averted	Cost-saving	Cost-saving	Cost-saving
**Azithromycin effectiveness in reducing infection (without sepsis or death)** [c]			
	**Base case azithromycin effectiveness (RR 0·75)**	**High azithromycin effectiveness (RR 0·66)**	**Low azithromycin effectiveness (RR 0·86)**
**Parameters adjusted (95% CI)**	··	··	··
Cases of infection (without sepsis or death) averted per 100,000 pregnancies	811·4 (460·7, 1136·2)	1126·7 (1029·6, 1227·7)	463·9 (424·0, 505·5)
**Cost-effectiveness outcome, USD (95% CI)**			
Incremental cost per 100,000 pregnancies	-32,661 (-118,210, 52,218)	-46,524 (-130,204, 35,715)	-17,382 (-102,859, 67,126)
Cost per case of infection, sepsis, or death averted	Cost-saving	Cost-saving	Cost-saving
Cost per case of sepsis or death averted	Cost-saving	Cost-saving	Cost-saving
Cost per case of infection (without sepsis or death) averted	Cost-saving	Cost-saving	Cost-saving
Cost per DALY averted	Cost-saving	Cost-saving	Cost-saving

Model results are based on the parameters in Table 1 and reflect the overall average healthcare use cost data. Negative dollar values represent a net savings.

[a] We based low and high azithromycin cost estimates on the low ($0·15/tablet) and high ($0·37/tablet) price of azithromycin from the 2015 International Medical Products Price Guide and calculated for a 2 g treatment regimen. Intervention cost values for the low and high scenarios were entered as constant and thus no CI is reported.

[b] Intervention cost not exactly equal to $0·91 per pregnancy due to stochastic variation across the 100,000 samples.

[c] Low and high azithromycin effectiveness estimates were based on upper and lower 95% CI reported in the A-PLUS trial (Tita et al., 2023).

Abbreviations: CI=confidence interval, USD=United States dollars, DALY=disability-adjusted life year, RR=relative risk, YLD=years lived with disability.

***Supplemental Figure 1:* Incremental cost-effectiveness ratio scatterplot for overall cost-effectiveness results of intrapartum azithromycin**

The cost per case of infection, sepsis, or death averted reflects parameters from Table 1 and the overall average healthcare use cost data. The figure shows 10,000 data points reflecting the incremental costs and effects from 100,000 model simulations. Each model simulation uses a different set of parameters randomly drawn from the distributions defined for each model parameter.

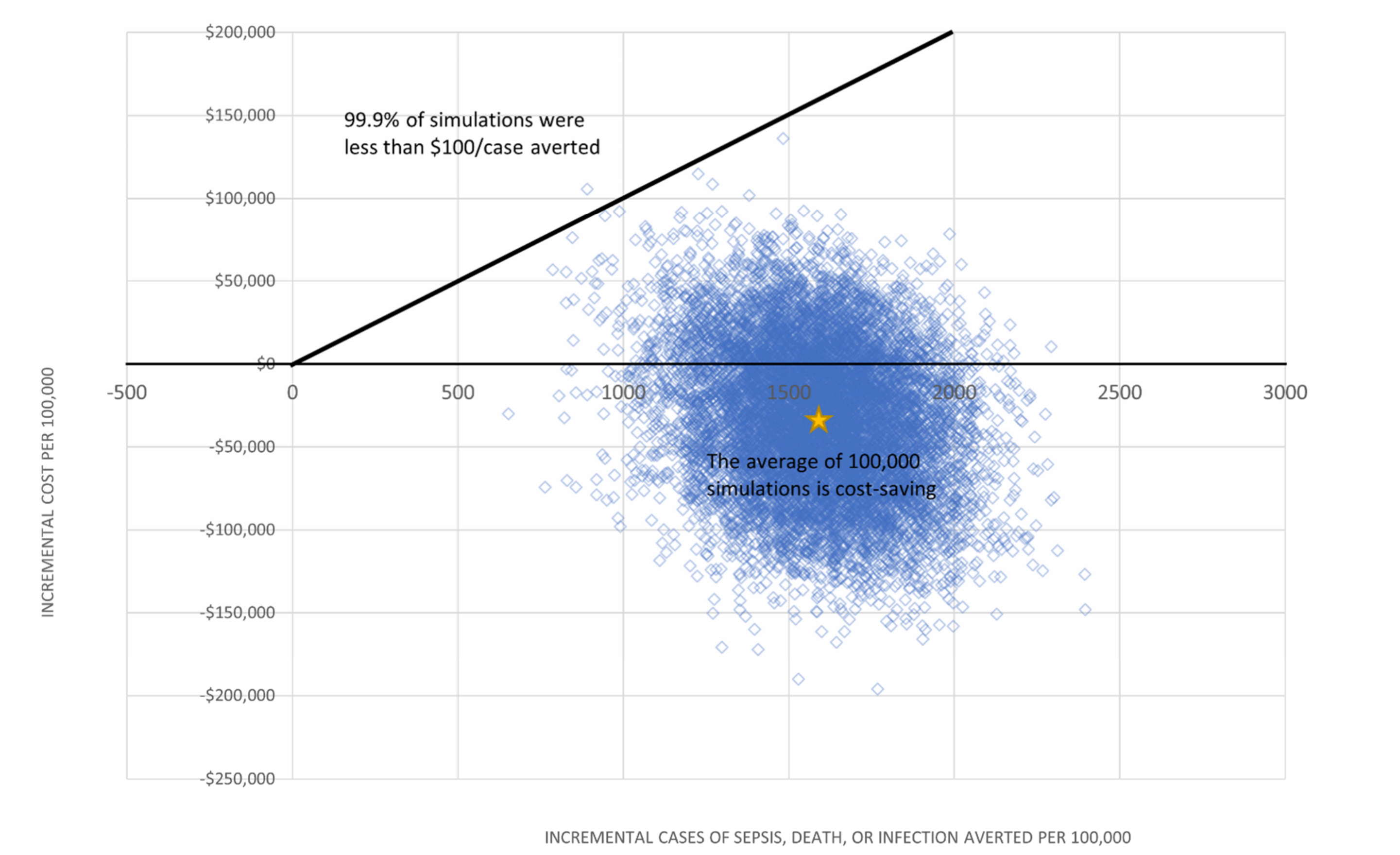

Supplement: Supplementary appendix 3 [file mmc3.pdf]
