# Supplementary material for: Cost-effectiveness of intrapartum azithromycin to prevent maternal infection, sepsis, or death in low-income and middle-income countries: a modelling analysis of data from a randomised, multicentre, placebo-controlled trial
Source: Lancet Glob Health. 2025 Mar 26;13(4):e679–88. doi: 10.1016/S2214-109X(24)00517-5 (PMC11950424; doi:10.1016/S2214-109X(24)00517-5)
Supplement: Equitable Partnership Declaration [file mmc4.pdf]

THE LANCET
Global Health

Supplementary appendix 4

This Equitable Partnership Declaration (EPD) was submitted by the authors, and we reproduce it as supplied. It has not been peer reviewed. *The Lancet*'s editorial processes have not been applied to the EPD.

Supplement to: Patterson JK, Neuwahl S, Kirsch S, et al. Cost-effectiveness of intrapartum azithromycin to prevent maternal infection, sepsis, or death in low-income and middle-income countries: a modelling analysis of data from a randomised, multicentre, placebo-controlled trial. *Lancet Glob Health* 2025; **13:** e679–88.

Equitable Partnership Declaration questions

This Equitable Partnership Declaration is a statement being published online alongside papers at *The Lancet Global Health*, as a separate appendix, to allow researchers to describe how their work engages with researchers, communities, and environments in the countries of study. This is part of our broader goal to decolonise global health, handing control and leadership of research to academics and clinicians who are based in the regions of study, and to affected communities.

Please answer all questions with as much detail as possible, noting that all included information will be published open-access and it will be freely available online to all who wish to read it. If a question does not apply to your study, please state "Not applicable".

The format of and questions in this statement are currently in a pilot phase. Please email Dr Liam Messin (Liam.Messin@lancet.com; deputy editor) and Dr Kate McIntosh (Kate.McIntosh@lancet.com; senior editor) with any feedback, particularly if you find any questions unclear.

Researcher considerations

1. Please detail the involvement that researchers who are based in the region(s) of study had during a) study design; b) clinical study processes, such as processing blood samples, prescribing medication, or patient recruitment; c) data interpretation; and d) manuscript preparation, commenting on all aspects. If they were not involved in any of these aspects, please explain why.

This question is intended for international partnerships; if all your authors are based in the area of study, this question is not applicable.

This should include a thorough description of their leadership role(s) in the study. Are local researchers named in the author list or the acknowledgements, or are they not mentioned at all (and, if not, why)? Please also describe the involvement of early career researchers based in the location of the study. Some of this information might be repeated from the Contributors section in the manuscript. Note: we adhere to ICMJE authorship criteria when deciding who should be named on a paper.

The *Eunice Kennedy Shriver's* National Institute of Child Health and Human Development's Global Network for Women's and Children's Health Research (NICHD GN) is a network of international research units in low- and middle-income countries (LMICs) and a data coordinating center. Each research unit is co-led by a US-based principal investigator (PI) and an international PI based at the research unit, with implementation of research studies overseen by an in-country coordinator and additional local study staff. In the responses below, we provide general information regarding the involvement of local investigators in all aspects of NICHD GN studies.<br><br>The Azithromycin Prevention for Labor Use (A-PLUS) study was conducted by the NICHD GN. This cost-effectiveness analysis of the A-PLUS trial is led by the same investigators as the main trial. When applicable, we provide specific examples relevant to the A-PLUS trial and this secondary cost-effectiveness analysis in the responses below.
**a) Study design:** International PIs and in-country coordinators are integrally involved in the design of each NICHD GN study, providing critical input on such elements as feasibility of conducting the

study in their low-resource environment and generalizability of the findings to other LMIC communities.
**b) Clinical study processes:** For all NICHD GN studies, local investigators sensitize communities to the study, recruit and enroll participants, administer the study intervention when applicable and conduct all follow-up. Specifically for this cost-effectiveness analysis, local investigators for each region of the study sought and provided cost data relevant for their region.
**c) Data interpretation:** The international PI and country coordinator of each research unit provide feedback on interpretation and presentation of NICHD GN studies including during semi-annual NICHD GN Steering Committee meetings and via email.
**d) Manuscript preparation:** All international PIs and country coordinators were included as co-authors of this cost-effectiveness analysis; each co-author reviewed and revised the manuscript.

2. Were the data used in your study collected by authors named on the paper, or have they been extracted from a source such as a national survey? ie, is this a secondary analysis of data that were not collected by the authors of this paper. If the authors of this paper were not involved in data collection, how were data interpreted with sufficient contextual knowledge?

   The Lancet Global Health *believe contextual understanding is crucial for informed data analysis and interpretation.*

The NICHD GN authors of the A-PLUS trial conducted this secondary cost-effectiveness analysis.

3. How was funding used to remunerate and enhance the skills of researchers and institutions based in the area(s) of study? And how was funding used to improve research infrastructure in the area of study?

   Potentially effective investments into long-term skills and opportunities within institutions could include training or mentorship in analytical techniques and manuscript writing, opportunities to lead all or specific aspects of the study, financial remuneration rather than requiring volunteers, and other professional development and educational opportunities.

   Improvements to research infrastructure could be funding of extended trial designs (such as platform trials) and use of master protocols to enable these designs, establishment of long-term contracts for research staff, building research facilities, and local control of funding allocation.

**Skills:** The A-PLUS trial built capacity at many of the participating research sites by teaching staff laboratory techniques including how to draw sterile cultures from mothers and newborns. All staff who participated in local implementation of the trial were paid staff contributing to the local infrastructure for clinical, public health and implementation research.
**Research infrastructure:** The NICHD GN provides support for local research infrastructure with protected effort for a US-based PI, an international PI and a country coordinator. This funding enables long-term contracts with research staff in-country beyond the life of an individual trial, and extending for the full period of the NICHD GN grant (typically five to seven years). NICHD GN trials benefit from this research infrastructure, and local investigators in each region determine how to allocate allotted funds to execute a given study.

4. How did you safeguard the researchers who implemented the study?

Please describe how you guaranteed safe working conditions for study staff, including provision of appropriate personal protective equipment, protection from violence, and prevention of overworking.

The international PI of each NICHD GN research unit ensures context-specific safe working conditions for all study staff. This includes ensuring that study budgets are sufficient for the work to be performed under safe working conditions.

Benefits to the communities and regions of study

5. How does the study address the research and policy priorities of its location?

How were the local priorities determined and then used to inform the research question? Who decided which priorities to take forward? Which elements of the study address those priorities?

The NICHD GN steering committee is composed of both US-based PIs and international PIs, and reviews study proposals and prioritizes them based on their public health relevance at each of the research units. In a formal voting process, the members of the steering committee collectively agree upon which priorities to advance.<br><br>Maternal mortality is high in LMICs and maternal mortality from sepsis among the leading causes of death. Given the magnamity of this public health threat, this study was a high research priority for all NICHD GN research units.

6. How will research products be shared in the community of study?

For instance, will you be providing written or oral layperson summaries for non-academic information sharing? Will study data be made available to institutions in the region(s) of study? The Lancet Global Health *encourages authors to translate the summary (abstract) into relevant languages after paper editing; do you intend to translate your summary?*

NICHD GN international PIs are responsible to lead dissemination of findings of all NICHD GN studies to their local communicities. Local strategies for dissemination may include meetings with Ministries of Health, local stakeholders and frontline health care providers.<br><br>Dissemination meetings will be held in all NICHD GN research sites to explain the results of this cost-effectiveness analysis. We will translate our summary into French and Spanish to ensure the results are readily available to individuals in the regions represented in the NICHD GN.

7. How were individuals, communities, and environments protected from harm?

a) *How did you ensure that sensitive patient data was handled safely and respectfully? Was there any potential for stigma or discrimination against participants arising from any of the procedures or outcomes of the study?*

All NICHD GN study protocols include a plan for protection of the data per the data coordinating center's recommendation and regulatory requirements. This includes assigning participants study IDs and attributing all data from a given participant to this ID. Each GN research unit adapts the approved data protection plan to fit the local context.

For the A-PLUS trial, all biologic specimens were labelled by the participant ID and thus de-identified. None of the procedures or outcomes of the study carried more than minimal potential for stigma or discrimination.

b) *Might any of the tests be experienced as invasive or culturally insensitive?*

No

c) *How did you determine that work was sensitive to traditions, restrictions, and considerations of all cultural and religious groups in the study population?*

As part of preparatory activities for any NICHD GN study, in-country coordinators sensitize local communities to the study. If previously unknown cultural or religious considerations are identified during community sensitization, these are addressed prior to study launch.

The cost effectiveness analyses did not add additional cultural or religious burden to the study.

d) *Were biowaste and radioactive waste disposed of in accordance with local laws?*

Yes for the main trial. Each research unit made a local plan for appropriate waste disposal in accordance with local laws.

e) *Were any structures built that would have impacted members of the community or the environment (such as handwashing facilities in a public space)? If so, how did you ensure that you had appropriate community buy-in?*

Not applicable

f) *How might the study have impacted existing health-care resources (such as staff workloads, use of equipment that is typically employed elsewhere, or reallocation of public funds)?*

For all NICHD GN Studies, each research unit develops a local plan for implementation that relies on paid study staff. NICHD GN studies frequently build capacity in local health facilities by providing equipment. No public funds from the international unit are used to support NICHD GN studies.

8. Finally, please provide the title (eg, Dr/Prof, Mr/Mrs/Ms/Mx), name, and email address of an author who can be contacted about this statement. This can be the corresponding author.

Name: Jackie Patterson

Email: jackie_patterson@med.unc.edu
